# The relationship between sex hormones, the vaginal microbiome and immunity in HIV-1 susceptibility in women

**DOI:** 10.1242/dmm.035147

**Published:** 2018-08-28

**Authors:** Jocelyn M. Wessels, Allison M. Felker, Haley A. Dupont, Charu Kaushic

**Affiliations:** 1 McMaster Immunology Research Centre, Department of Pathology and Molecular Medicine, Michael G. DeGroote Centre for Learning and Discovery, McMaster University, Hamilton, Ontario L8S 4L8, Canada; 2 Department of Pathology and Molecular Medicine, McMaster University, Hamilton, Ontario L8S 4L8, Canada

**Keywords:** Vaginal microbiota, T cells, DMPA, Inflammation

## Abstract

The role of sex hormones in regulating immune responses in the female genital tract has been recognized for decades. More recently, it has become increasingly clear that sex hormones regulate susceptibility to sexually transmitted infections through direct and indirect mechanisms involving inflammation and immune responses. The reproductive cycle can influence simian/human immunodeficiency virus (SHIV) infections in primates and HIV-1 infection in *ex vivo* cervical tissues from women. Exogenous hormones, such as those found in hormonal contraceptives, have come under intense scrutiny because of the increased susceptibility to sexually transmitted infections seen in women using medroxyprogesterone acetate, a synthetic progestin-based contraceptive. Recent meta-analyses concluded that medroxyprogesterone acetate enhanced HIV-1 susceptibility in women by 40%. In contrast, estradiol-containing hormonal contraceptives were not associated with increased susceptibility and some studies reported a protective effect of estrogen on HIV/SIV infection, although the underlying mechanisms remain incompletely understood. Recent studies describe a key role for the vaginal microbiota in determining susceptibility to sexually transmitted infections, including HIV-1. While *Lactobacillus* spp.-dominated vaginal microbiota is associated with decreased susceptibility, complex microbiota, such as those seen in bacterial vaginosis, correlates with increased susceptibility to HIV-1. Interestingly, sex hormones are inherently linked to microbiota regulation in the vaginal tract. Estrogen has been postulated to play a key role in establishing a *Lactobacillus*-dominated microenvironment, whereas medroxyprogesterone acetate is linked to hypo-estrogenic effects. The aim of this Review is to contribute to a better understanding of the sex-hormone–microbiome–immunity axis, which can provide key information on the determinants of HIV-1 susceptibility in the female genital tract and, consequently, inform HIV-1 prevention strategies.

## Abstract (precis)

**Summary:** A better understanding of the sex-hormone–microbiome–immunity axis can provide key information about the determinants of HIV-1 susceptibility in the female genital tract and consequently inform HIV-1 prevention strategies.

## Introduction

Clinical and experimental evidence indicates that many sexually transmitted infections (STIs) are more prevalent in women than men (Kaushic et al., 2011). The probability of human immunodeficiency virus (HIV) transmission via the female genital tract (FGT) is approximately 1.5- to 5-times greater than via the male genital tract (1 in 2000 to 1 in 200 in females versus 1 in 3000 to 1 in 700 in males) (Hladik and McElrath, 2008). There are both socio-economic and biological reasons why women may be more susceptible to STIs, including HIV-1, than men. The biological factors that could influence the outcome of pathogen exposure in the FGT include its large surface area, the alterations in physiology of reproductive tract tissues during different phases of the menstrual cycle, the influence of sex hormones on mucosal immune defense, the use of hormonal contraceptives and the effect of the indigenous microbiota (see Box 1 for a glossary of terms). In this Review, we highlight the mechanisms by which sex steroid hormones, including hormonal contraceptives, might impact the risk of HIV-1 susceptibility in women. This is an important and timely topic, given that approximately 40% of HIV-1 infections occur in the FGT and that women using the progestin-based injectable contraceptive depot-medroxyprogesterone acetate (DMPA) are 40% more likely to acquire HIV-1 than women not using hormonal contraceptives (Polis et al., 2016). The relevance of this area to public health is emphasized by the fact that more than 8 million women in sub-Saharan Africa, where HIV-1 is endemic, use DMPA as their main form of contraception (Ross and Agwanda, 2012).

**Box. 1. Glossary**

**16S rRNA gene sequencing:** an untargeted method to identify the bacterial taxa present in a microbiota. Typically, a variable region within the 16S ribosomal RNA (rRNA) gene that is unique to each taxa and codes for a component of the bacterial ribosome is amplified by PCR and sequenced.

**Alpha-diversity:** a term used in ecology to describe differences in species within a site, on a ‘local’ scale. In the context of this article, we are referring to the unique diversity of bacterial species found in the vaginal microbiota of each woman in our study.

**Amsel criteria:** a method to diagnose bacterial vaginosis (BV). Diagnosed by the presence of at least three of four clinical symptoms [thin/white/yellow vaginal discharge, clue cells (vaginal epithelial cells that appear stippled owing to being covered with bacteria) on microscopy, vaginal pH>4.5, fishy odor following addition of potassium hydroxide]. The Amsel criteria distinguish nonspecific vaginitis (BV) from other forms of vaginitis and from normal findings, as defined in Amsel et al. (1983).

**Antimicrobial peptides (AMPs):** part of the innate immune response for host defense, found in all classes of life.

**Cervical transformation zone:** the area of the cervix where the columnar epithelial cells lining the endocervix change into the squamous epithelial cells lining the ectocervix; an area thought to be particularly prone to HIV-1 acquisition.

**Commensal:** an organism living in relationship with another without harming or benefitting the host organism.

**Defensins:** small proteins found in vertebrates and invertebrates that function as antimicrobial host defense peptides.

**Dysbiosis:** a microbial imbalance.

**Estradiol** **(E2)-based hormone replacement therapy (HRT):** treatment with natural or synthetic estrogens alone or in combination with progestins, typically aimed at alleviating the symptoms of menopause, or preventing osteoporosis.

**Ectocervix:** the outer portion of the cervix that is located within the vaginal tract and lined by squamous epithelial cells.

**Elafin:** a defensin (antimicrobial peptide) with antibacterial activity against bacterial and fungal pathogens. Also known as Trappin-2.

**HIV-1 gp120:** a glycoprotein found on the exposed surface of the HIV-1 envelope.

**Lactoferrin:** a defensin (antimicrobial peptide) that also binds iron.

**Lysozyme:** a defensin (antimicrobial protein) that enzymatically cleaves and thus damages the components of the cell wall of Gram-positive bacteria.

**Microbiota:** a consortium of bacteria residing in and on multicellular organisms, including plants and animals.

**Mucins:** heavily glycosylated proteins (glycoproteins) produced by epithelial cells in most animals. They are a constituent of vaginal mucus.

**NOD-like receptors:** intracellular sensors of pathogen-associated molecular patterns (PAMPs). A type of pattern recognition receptor (PRR).

**Pattern recognition receptors (PRRs):** part of the innate immune system, these protein sensors detect pathogen-associated molecular patterns (PAMPs) and induce an innate response in the host.

**RANTES (also known as CCL5):** stands for ‘regulated on activation, normal T cell expressed and secreted’. A chemotactic protein (chemokine) that recruits T cells, eosinophils and basophils, or other cells with cognate receptors.

**Seroconversion:** the point at which a specific antibody has developed and become detectable in the peripheral blood (i.e. antibodies against HIV-1 indicate that seroconversion has occurred).

**Seronegative:** a negative result in a blood test for antibodies against a certain virus or condition (i.e. HIV-1 or rheumatoid arthritis).

**Toll-like receptors (TLRs):** intra- or extracellular proteins that recognize microbes and nucleic acids, and participate in the innate immune response. A type of pattern recognition receptor (PRR).

## The female genital tract

The lower FGT, the vaginal tract and ectocervix (Box 1), is lined with epithelial cells covered by mucus and colonized by bacteria. It is the first location encountered by HIV-1 during heterosexual intercourse with an infected male partner. The lower FGT provides a protective physical and immunological mucosal barrier. Acting as a structural support beneath the epithelium is a dense layer of stromal fibroblasts, in which a diverse population of leukocytes reside (Wira et al., 2005). As HIV-1 preferentially infects CD4^+^ leukocytes [T cells, macrophages and dendritic cells (DCs)] residing in the stroma, the vaginal mucus and epithelial barrier serve in part to impede viral access to target cells (Pope and Haase, 2003). In order for HIV-1 transmission to occur, infectious virions must transverse the protective physical and immunological barriers of the FGT and infect target cells located in the stroma. While the exact mechanisms by which HIV-1 accesses target cells remain incompletely understood, this Review will focus on several factors known to influence HIV-1 susceptibility and acquisition in the FGT.

The lower FGT is lined with multi-layered squamous epithelial cells, and tight junctions linking these cells are mainly restricted to its basal layers. The epithelium in the lower FGT undergoes continuous differentiation, resulting in a mitotically active basal layer and a terminally differentiated superficial layer comprising cornified epithelial cells, which aid in preventing infection by certain pathogens, including HIV-1 (Anderson et al., 2014). Aside from acting as a physical barrier, the vaginal epithelial cells also secrete mucins (Box 1) into the vaginal lumen. These form a hydrophobic layer of mucus that traps pathogens and prevents access to the underlying epithelial cells (Lai et al., 2009; Quayle, 2002; Shukair et al., 2013). In addition to mucins, the vaginal mucus contains other host defense molecules, including antimicrobial peptides (AMPs; Box 1) and complement system components, which can directly bind to and eliminate pathogens, impeding access to the vaginal epithelium (Birse et al., 2015b; Quayle, 2002). These protective features can effectively dampen HIV-1 motility in human cervicovaginal mucus and enhance vaginal barrier function (Shukair et al., 2013). Colonizing the vaginal epithelium is an indigenous microbial community that can influence the physiology and immune function of the FGT (Cruickshank and Sharman, 1934; Ma et al., 2012). The resident vaginal microbiota (VMB) exists in a mutualistic relationship with the female host and also participates in preventing vaginal infection by a variety of pathogens. Notably, disruption of the VMB is associated with an increased risk of STIs, including HIV-1 (Atashili et al., 2008). The impact of the VMB on inflammation and susceptibility to HIV-1 is an emerging area of interest and will be discussed in more detail below.

The upper FGT consists of the endocervix, uterus (endometrium), fallopian tubes and ovaries. It is lined by a single layer of epithelial cells linked by tight junctions that provide physical protection. It was originally believed that the upper FGT was sterile. However, like the vaginal tract, the upper FGT is colonized by bacteria (Chen et al., 2017; Eschenbach et al., 1986; Hemsell et al., 1989; Mitchell et al., 2015; Møller et al., 1995). Several studies have reported significant correlation in microbial community members across the FGT, but their relative proportions vary (Chen et al., 2017; Walther-António et al., 2016; Wee et al., 2018). Furthermore, the quantity of bacteria found on the endometrial surface are 2-4 log orders of magnitude lower than those found in the vaginal tract (Chen et al., 2017; Mitchell et al., 2015), and lactobacilli typically represent a large fraction of the endometrial microbiota (Franasiak et al., 2016; Mitchell et al., 2015; Moreno et al., 2016; Wee et al., 2018). However, studies also identified many low-abundance genera (Moreno et al., 2016; Verstraelen et al., 2016). Similar to the VMB, the endometrial microbiota may be able to modulate inflammation. *In vitro* co-cultures of endometrial epithelial cells with pathogenic bacteria (*Neisseria gonorrhoeae*) induced proinflammatory mediators (Christodoulides et al., 2000), whereas other bacteria typically found in the reproductive microbiotas (*Lactobacillus crispatus*, *Gardnerella vaginalis*) did not (Łaniewski et al., 2017). This suggests that the endometrial microbiota may be able to modulate endometrial inflammation in the host. HIV-1 infection can occur in the upper FGT, although the exact mechanism is unclear. HIV-1 has been shown to induce innate inflammation that can disrupt the mucosal barrier in the upper FGT (Nazli et al., 2010), to transcytose across the endometrial epithelial barrier (Ferreira et al., 2015; Hocini et al., 2001) and to infect ovarian tissue cells *in vitro* (Shen et al., 2017), which could influence initiation of HIV-1 infection. Simian immunodeficiency virus (SIV), the primate analog of HIV, can also rapidly access and infect target cells throughout the lower and upper FGT (Stieh et al., 2014). A number of other reviews have focused on the possible mechanisms of HIV-1 infection in the upper FGT (Dunbar et al., 2012; Ferreira et al., 2014; Hickey et al., 2011; Lee et al., 2015; Nguyen et al., 2014; Wira et al., 2015). In this Review, we discuss the recent information that is relevant to HIV-1 transmission in the lower FGT (ectocervix and vagina), which make up the major surface area exposed to HIV-1-infected semen during heterosexual intercourse.

Although one of the main functions of the immune system in the FGT is to protect against an array of pathogens, it must concurrently allow for the main reproductive functions of the FGT, including supporting sperm migration, oocyte fertilization and embryo implantation. To balance these unique requirements, the FGT is precisely regulated by the sex steroid hormones estradiol (E2) and progesterone, which are cyclically produced by the ovaries throughout the menstrual cycle during the reproductive years in women (Reed and Carr, 2000; Wira et al., 2005, 2015). Both fluctuating endogenous sex hormones and hormonal contraceptives can alter the components of the FGT defensive barriers, including mucus viscosity, epithelial barrier thickness, immune cell frequency and resident vaginal microbes (Vitali et al., 2017; Wira et al., 2015). For example, the amount and composition of the vaginal mucus varies with the menstrual cycle, hormonal contraceptive use, and hormone dampening at menopause (Chappell et al., 2015; Gipson et al., 1997; Shust et al., 2010). During ovulation, under the influence of estradiol, the vaginal mucus is thin with low viscosity, which facilitates sperm movement. Conversely, during the progesterone-high luteal phase of the menstrual cycle, the vaginal mucus is thick and viscous, which impedes the movement of particulates from the lower to the upper FGT. Together, the carefully regulated fluctuations of these physicochemical features are necessary to promote both reproductive success and a robust defensive barrier in the FGT. As these first lines of defense are heavily influenced by both hormones and microbiota composition, their dynamic interactions can substantially influence HIV-1 susceptibility in women, and will be discussed in detail below.

## Immunity in the lower female genital tract

**Fig. 1.**
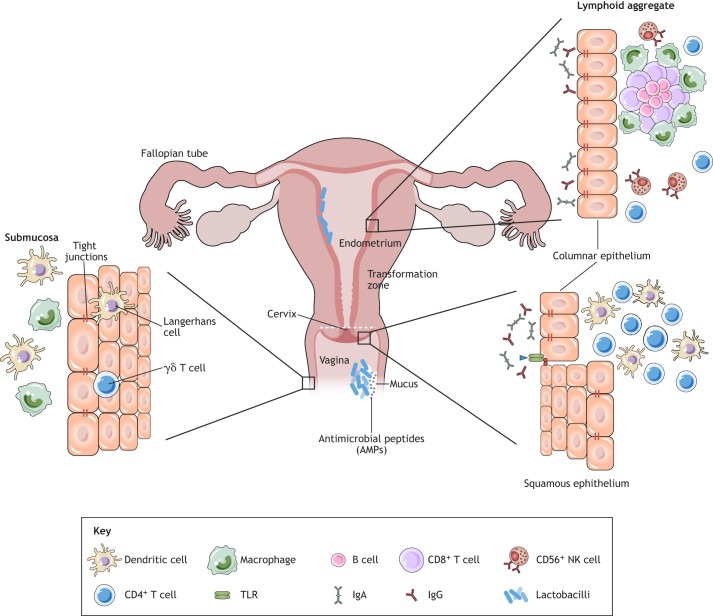
**Anatomy and immunological components of the female genital tract.** The female genital tract (FGT) can be separated into the upper (ovary, fallopian tube, uterus/endometrium and endocervix) and lower (ectocervix and vagina) tract. The vaginal epithelium has many innate immune protection mechanisms, such as tight junctions, antimicrobial peptides (AMPs) and mucus, in order to neutralize, trap and prevent entry of potential pathogens. The vaginal lumen is colonized by commensal bacteria, mainly lactobacilli, which help to maintain a low pH. Furthermore, immune cells such as γδ T cells, dendritic cells (DCs) and macrophages are present beneath and between the vaginal epithelial cell layer to survey the local environment for danger. The abrupt transition from keratinized squamous epithelial cells of the ectocervix to single columnar epithelial cells of the endocervix represents the transformation zone; this site has an abundance of HIV-1 target cells and has been proposed to be one of the major sites for infections. The presence of lymphoid aggregates in the endometrial tissue suggests that this is an inductive site for cell-mediated immunity. Lymphoid aggregates found beneath the endometrium are composed of B cells in the inner core surrounded by CD8^+^ CD4^−^ T cells and an outer layer of macrophages. A scatter of CD56^+^ natural killer (NK) cells and CD4^+^ T cells could be found in between lymphoid aggregates. It was originally believed that the upper FGT was sterile; however, like the vaginal tract, the upper FGT is colonized by bacteria, including lactobacilli. Figure modified and reprinted with permission from Nguyen et al. (2014). TLR, Toll-like receptor.

In the lower FGT, epithelial cells, stromal fibroblasts and leukocytes interact with each other, with the sex hormones and with the VMB to induce the two arms of the immune response: innate and adaptive immunity (Ferreira et al., 2014; Nguyen et al., 2014; Zhou et al., 2018). In addition to acting as a physical barrier against pathogens, the epithelial cells of the lower FGT modulate leukocyte function by producing cytokines and chemokines, which induce leukocyte differentiation and mediate inflammatory processes (Fahey et al., 2005). Epithelial cells in both the upper and lower FGT (Fig. 1) possess a wide array of pattern recognition receptors (PRRs), including Toll-like receptors (TLRs) and NOD-like receptors (Box 1), which recognize and respond to pathogens through conserved pathogen-associated molecular patterns (PAMPs) expressed by microorganisms (Ghosh et al., 2013; Herbst-Kralovetz et al., 2008; Pioli et al., 2004). However, PRRs appear to be differentially regulated and expressed by epithelial cells depending on their location within the FGT, with a limited expression of PRRs in the lower compared to the upper FGT (Ghosh et al., 2013; Hart et al., 2009; Pioli et al., 2004). Pathogen recognition by PRRs induces intracellular signaling and activates the transcription factor NF-κB, resulting in the production of AMPs and pro-inflammatory cytokines (Fichorova et al., 2002; Nguyen et al., 2014). For instance, primary epithelial cells isolated from the human vagina and ectocervix can induce NF-κB signaling upon activation of the TLR pathway to produce pro-inflammatory cytokines, including interleukin 6 (IL-6), IL-8, tumor necrosis factor α (TNF-α), macrophage inflammatory protein 1α (MIP-1α) and MIP-1β (Fichorova et al., 2015, 2002). Indeed, epithelial cells from both the upper and lower FGT have been reported to activate signaling pathways following pathogen exposure (Ferreira et al., 2013; Nazli et al., 2010, 2009). Furthermore, our group recently demonstrated that HIV-1 gp120 (Box 1) signaling through TLR2 induced interferon β (IFN-β) production in genital epithelial cells, which had an anti-inflammatory activity and protected the function of the epithelial barrier (Nazli et al., 2018). In addition to inflammatory cytokines, cervicovaginal epithelial cells are known to produce AMPs, including defensins (Box 1), secretory leukocyte protease inhibitor (SLPI), lysozyme, lactoferrin and elafin (Box 1) (Nguyen et al., 2014; Wira et al., 2005). *In vitro* cultures of ectocervical epithelial cells secrete AMPs, including SLPI1, Trappin-2/elafin and human beta defensin 2 (HBD-2) (Wira et al., 2011), which are known to have anti-HIV-1 activity (Aboud et al., 2014; Ghosh et al., 2010; McNeely et al., 1995; Sun et al., 2005). Taken together, the epithelial cells of the lower FGT contribute to recognition of and defense against pathogens in part via their induction of inflammation and AMPs.

Residing in the lower FGT are numerous, dynamic leukocyte populations that contribute to humoral and cellular immunity. Leukocytes constitute 6-20% of all cells in the FGT (Nguyen et al., 2014; Wira et al., 2015), and possess unique characteristics that differentiate them from other tissue resident and peripheral leukocytes. For instance, flow cytometry measurements showed that primary vaginal macrophages were more susceptible to HIV-1 infection *in vitro* than gastrointestinal macrophages (Shen et al., 2011). Leukocytes of the FGT are preferentially distributed in immunological microenvironments depending on their function within the FGT. In the cervical transformation zone (Box 1), T cells and antigen-presenting cells (APCs), including many CD14^+^ macrophages and DCs, are particularly abundant (Pudney et al., 2005; Trifonova et al., 2014). The presence of CD4^+^CCR5^+^ T cells, which are the primary targets for HIV-1 infection, renders the transformation zone particularly vulnerable (Eslahpazir et al., 2008; Kumamoto and Iwasaki, 2012; Pudney et al., 2005; Trifonova et al., 2014; Vitali et al., 2017). DCs, including intra-epithelial CD1a^+^ Langerhans cells, are present in the squamous epithelial cell layers of the ectocervix and vagina (Pudney et al., 2005). These cells can be infected by and are thought to facilitate HIV-1 transfer across epithelial cells of the lower FGT (Ballweber et al., 2011; Tchou et al., 2001). Further, cervical DCs express DC-SIGN, a C-type lectin receptor and adhesion molecule that also acts as an HIV-1 receptor. The presence of DC-SIGN on DCs has been implicated in enhanced transmission of HIV-1 to T cells (Geijtenbeek et al., 2000; Geijtenbeek and van Kooyk, 2003). Thus, while T cells are the main targets for HIV-1, macrophages and DCs can also be infected and therefore directly and indirectly affect HIV-1 susceptibility in women.

Most T cells in the lower FGT reside near the basal epithelial cell layer at the stromal cell interface; however, a large T-cell population also resides in the ectocervical and vaginal epithelium as intra-epithelial lymphocytes (Pudney et al., 2005). Although CD8^+^ T cells are slightly more abundant than CD4^+^ T cells (Trifonova et al., 2014), the CD4^+^ T cells represent a greater number of the intra-epithelial lymphocytes in the ectocervix (Pudney et al., 2005). Interestingly, the number of vaginal intra-epithelial CD4^+^ and CD8^+^ T cells, as well as CD1a^+^ DCs, is elevated in women with inflammation of the FGT (Pudney et al., 2005), and CD8^+^ T cells of the vagina and cervix possess cytolytic activity (Pudney et al., 2005; White et al., 1997a). In recent years, T-helper 17 (Th17) cells have been identified as preferential targets for HIV-1 in the human cervix (Cicala et al., 2009; McKinnon et al., 2011, 2015). These CD4^+^IL17A^+^ cells co-express the HIV-1 co-receptor CCR5 and mucosal integrin α4β7, which is known to bind HIV-1 gp120 and enhance viral dissemination (Cicala et al., 2009). Importantly, this Th17 population is almost completely depleted in HIV-1^+^ women, indicating its potential as a key target for HIV-1 transmission (McKinnon et al., 2011). Thus, given their abundance, susceptibility and location, CD4^+^ T and Th17 cells are considered the major targets of HIV-1 in the lower FGT. Hence, the factors influencing the number and activation of these cells can greatly impact HIV-1 susceptibility.

**Fig. 2.**
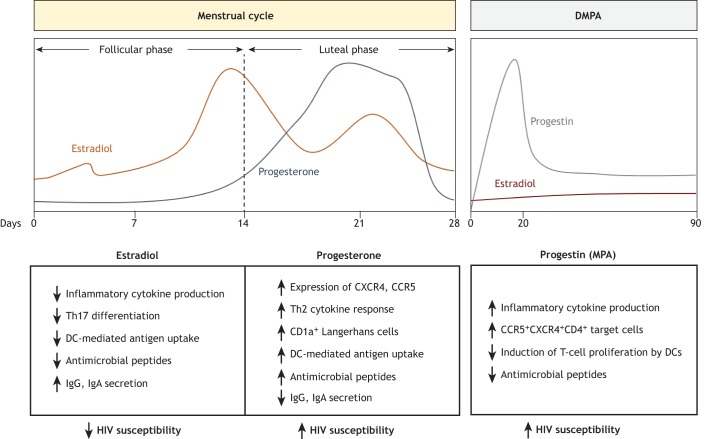
**Changes in lower female genital tract immunity and HIV-1 susceptibility under endogenous and exogenous sex hormones.** The endogenous levels of female sex hormones (estradiol and progesterone) vary throughout the 28-day menstrual cycle in women. Estradiol dominates the follicular phase and reaches peak levels just prior to ovulation, which occurs around day 14. Post-ovulation, estradiol levels decline as progesterone levels rise towards the mid-luteal phase. The progestin-based injectable contraceptive depot-medroxyprogesterone acetate (DMPA) is administered every 3 months. Levels of this progestin, a synthetic progesterone, are highest in serum within the first 20 days of administration. Women on DMPA have consistently lower estradiol levels than normally cycling women (Hapgood et al., 2018). A summary of the putative effects of hormones and hormonal contraceptives on female genital tract (FGT) immunity as presented in this Review are summarized as shown. Overall, while estradiol appears to promote factors related to decreased HIV-1 susceptibility, alterations in immunity during periods of high progesterone or DMPA use are associated with increased HIV-1 susceptibility. DCs, dendritic cells; Ig, immunoglobulin; Th2, T-helper type 2 cells; Th17, T-helper 17 cells.

Although B cells are a key cellular component of the adaptive immune system, they are not present in large numbers in the lower FGT. Resident immunoglobulin A (IgA)-expressing plasma cells have been described in the cervix, especially in the endocervix (Nguyen et al., 2014). In fact, studies examining antibody responses in the FGT have identified modest IgA and IgG responses against viral infections, including HIV-1, in cervical secretions (Russell and Mestecky, 2002). Unlike in other mucosal sites, the dominant antibody isoform produced during the adaptive immune response in the FGT is IgG rather than IgA (Li et al., 2011; Russell and Mestecky, 2002), yet both isoforms can transfer across the genital epithelium. In the case of IgG, the neonatal Fc receptor (FcRN) on genital epithelial cells facilitates transfer of IgG to the lumen to confer protection against vaginal infections (Li et al., 2011). Even though relatively little is known about the role of B cells and antibodies in the FGT, in theory they have the potential to contribute to adaptive immunity and offer an additional layer of protection against HIV-1. An overall summary of the general changes in immunity over the menstrual cycle are highlighted in Fig. 2.

## Effect of endogenous and exogenous sex steroid hormones on immunity in the FGT

It is well documented that the endogenous female sex steroid hormones estradiol and progesterone, and their synthetic analogs, including those found in hormonal contraceptives, regulate immunity in the FGT. Other reviews provide a detailed account of this immunoregulatory influence on immune cell phenotype and function (Dunbar et al., 2012; Kaushic, 2011; Nguyen et al., 2014; Vitali et al., 2017; Wira et al., 2014). In this article, we focus on the effect of endogenous and exogenous hormones in modulating key target cell populations, and thus HIV-1 susceptibility, in the lower FGT.

Although alterations in immune cell populations occur in endometrial tissues of the upper FGT (Nguyen et al., 2014), flow cytometry analyses did not confirm major fluctuations in their location or abundance in the lower FGT between the phases of the menstrual cycle or between pre- and post-menopausal women (Givan et al., 1997; Pudney et al., 2005; Trifonova et al., 2014). High levels of cytolytic activity were observed in CD3^+^ T cells isolated from the cervix and vagina, but this was independent of menstrual cycle stage. Women of reproductive age have significantly more CD4^+^, CD8^+^ and B cells in their ectocervix than in the endocervix (Trifonova et al., 2014). Further, the ectocervix has also been shown to contain higher numbers of CD4^+^ T cells in women with vaginal/cervical inflammation (Pudney et al., 2005; White et al., 1997b). However, other phenotypic and functional changes can occur within specific immune cell populations in response to sex hormones. For example, cellular proliferation and secretion of Th1-type cytokines was impaired in peripheral blood CD4^+^ and CD8^+^ T cells treated with progesterone, instead favoring the development of Th2-type cytokine-producing T cells (Enomoto et al., 2007; Piccinni et al., 1995). Progesterone treatment of murine bone-marrow-derived DCs resulted in inhibited differentiation of DCs, leading to increased antigen uptake and decreased production of inflammatory cytokines, whereas opposing effects were observed after estradiol treatment (Xiu et al., 2016). Furthermore, immunohistochemistry of vaginal biopsies from women of reproductive age who received intravaginal delivery of exogenous progesterone showed increased numbers of CD1a^+^ Langerhans cells (Wieser et al., 2001), which are implicated in HIV-1 transfer across epithelial cell barriers (Ballweber et al., 2011; Tchou et al., 2001). In addition to affecting HIV-1 target (CD4^+^HLA-DR^+^CD38^+^CCR5^+^) cells, progesterone also alters humoral immunity, as IgG and IgA titers in the cervical secretions were diminished in the luteal compared to the follicular phase of the menstrual cycle (Safaeian et al., 2009), and immunoglobulin levels peaked just prior to ovulation, when estradiol levels were high (Franklin and Kutteh, 1999; Kutteh et al., 1998). Similarly, progesterone is elevated in the luteal phase and is correlated with increased secretion of AMPs including defensins, trappin-2/elafin and SLPI in cervicovaginal lavage (Dunbar et al., 2012; Ghosh et al., 2010). Collectively, these results help illuminate the possible mechanisms by which progesterone might modulate immunity in the FGT and relate to HIV-1 susceptibility, which is greater in women in the progesterone-high luteal phase of the menstrual cycle (Saba et al., 2013; Wira and Fahey, 2008).

Although progesterone has been linked to increased HIV-1 susceptibility, estradiol can also impact immunity in the FGT. Depending on its concentration, estradiol can exert pro- or anti-inflammatory responses and differentially affect immune function (Straub, 2007). Both sex steroid hormones can also affect PRR expression throughout the FGT. Estradiol is able to modulate the downstream signaling of PRRs and pro-inflammatory receptors, as well as regulate NF-κB function (Fahey et al., 2008; Ghisletti et al., 2005; Schaefer et al., 2005). For instance, estradiol inhibits the mRNA expression of inflammatory cytokines IL-1α, Il-6, IL-8 and TNF-α in vaginal epithelial cell lines (Wagner and Johnson, 2012). Similarly, *in vitro* studies of human ectocervical epithelial cells treated with estradiol showed decreased production of IL-1β and IFN-γ (S Lashkari and Anumba, 2017), and vaginal cells captured using a menstrual cup and treated with estradiol had decreased secretion of the AMPs HBD2 and elafin (Patel et al., 2013). In primary murine microglia and RAW 264.7 cell macrophage populations, treatment with estradiol induced anti-inflammatory activity by inhibiting intracellular transport of NF-κB family members to the nucleus (Ghisletti et al., 2005), while exogenous estradiol treatment of peripheral blood macrophages and CD4^+^ T cells reduced HIV-1 viral entry through a CCR5-independent pathway (Rodriguez-Garcia et al., 2013). Estradiol can also inhibit *in vitro* Th17 differentiation in mouse splenocytes (Chen et al., 2015), and mouse models of viral infection have demonstrated that estradiol can enhance the response of Th17 cells and Th1-type responses following viral exposure (Anipindi et al., 2016). Taken together, these results suggest that estradiol typically maintains an anti-inflammatory immune environment that helps reduce susceptibility to infections while still maintaining the ability to mount an inflammatory, anti-viral response when necessary.

In addition to the impact of endogenous sex steroid hormones on immunity in the FGT, exogenous hormones, such as those commonly found in hormonal contraceptives, can also affect immunity in the FGT. This is an area of active research, particularly because the progestin-based injectable contraceptive DMPA is linked to increased HIV-1 susceptibility (Hapgood et al., 2018; Kaushic, 2009; Morrison et al., 2015; Polis et al., 2016). In a recent study, women on DMPA had greater expression of RANTES (also known as CCL5; Box 1) and decreased expression of the AMP BD2, which were implicated in HIV-1 seroconversion (Box 1) (Fichorova et al., 2015). Furthermore, an increased frequency of the HIV-1 target CCR5^+^CD4^+^ T cells was also observed in cervical cell populations isolated from women using injectable progestin-only contraceptives, including DMPA (Byrne et al., 2016; Chandra et al., 2013). In contrast, women using a progestin-based intrauterine device (IUD) had reduced expression of the HIV-1 co-receptor CCR5 on cervical T cells (Achilles et al., 2014). However, increased numbers of HIV-1 target cells are not consistently observed in women on DMPA (Michel et al., 2015; Mitchell et al., 2014). MPA, the active component in DMPA, was also shown to inhibit the activation of T cells and peripheral dendritic cells (pDCs) in response to T-cell-receptor- and TLR-mediated activation at physiological concentrations in peripheral blood mononuclear cells derived from pre-menopausal women (Huijbregts et al., 2014). In other studies, MPA also impaired the expression of DC activation markers and affected their ability to promote T-cell proliferation (Quispe Calla et al., 2015). Recent transcriptomic studies from our group investigated the impact of sex hormones and MPA on the mRNA profile of primary endometrial epithelial cells and found that MPA significantly increased expression of inflammation-related genes (Woods et al., 2018). Taken together, MPA is thought to negatively impact immunity as it relates to HIV-1 susceptibility in the FGT, despite several studies reporting it to be immunosuppressive (Hapgood et al., 2018). The overall changes in immunity under the influence of DMPA are highlighted in Fig. 2.

Combined oral contraceptives (COCs), which contain analogs of both estradiol and progesterone, are generally thought to have protective effects against viral infection in the FGT, depending on their formulations and quantity of the analogs. For example, one study found higher levels of inflammatory cytokines, including IL-1β, IL-6 and IL-8, in women on COCs compared to women who were not using hormonal contraceptives (Fichorova et al., 2015). While T cells isolated from the cervical epithelium of women using COCs showed no difference in expression of activation markers or CXCR4 compared to women not on COCs, the number of T cells expressing CCR5 was higher in women on COCs (Prakash et al., 2002). Conversely, multiple studies investigating immunoglobulins in cervical secretions revealed that women on COCs had higher IgG and IgA levels than naturally cycling women (Franklin and Kutteh, 1999; Safaeian et al., 2009), suggesting that adaptive immunity might be stronger in the women on COCs. Thus, exogenous exposure to hormonal contraceptives impacts immunity in the lower FGT, in ways that may impact susceptibility to HIV-1 infection.

## Factors affecting susceptibility to HIV-1 in women

To establish infection in the FGT, HIV-1 must first evade the previously discussed anatomical and biological barriers. Although these are effective, since the frequency of HIV-1 infection is approximately 1 in 200 to 1 in 2000 per act of unprotected receptive vaginal intercourse (Hladik and McElrath, 2008), several factors can influence susceptibility (Ferreira et al., 2014). Firstly, disruption of the vaginal epithelial barrier can enhance HIV-1 susceptibility in women. HIV-1 can penetrate the squamous epithelium of the lower FGT through simple diffusive percolation, and the depth of viral penetration significantly increases when the epithelial tight junctions are disrupted (Carias et al., 2013). Disruption of the vaginal epithelial barrier can occur during heterosexual intercourse as a result of microabrasions. These activate local inflammation and wound-healing processes, and promote infiltration of immune cells, which could ultimately amplify the population of HIV-1 target cells (Miller and Shattock, 2003; Norvell et al., 1984). In fact, women with disruptions of the genital epithelium are at the greatest risk of HIV-1 acquisition (hazard ratio of 4.30) compared to women without compromised epithelial barriers (Abbai et al., 2016), further supporting the association between barrier disruption and HIV-1 acquisition. Alternatively, the epithelial barrier can be disrupted by HIV-1 and its gp120, which disrupt tight-junction proteins like ZO-1 and occludin in cultured primary upper FGT cells (Nazli et al., 2010, 2013). *In vivo* studies have also shown that differing hormonal and microbial compositions influence protease levels in the FGT, which may in turn impact the integrity of the vaginal epithelial barrier and influence HIV-1 susceptibility (Arnold et al., 2016; Birse et al., 2015a; Borgdorff et al., 2016). Normally, the low pH of the vagina provides another layer of protection against pathogens. However, the alkaline pH of semen temporarily increases vaginal pH, and this can prevent HIV-1 from being neutralized by the normally acidic cervicovaginal mucus (Lai et al., 2009; Tevi-Bénissan et al., 1997), thereby increasing the risk of infection. Therefore, mechanisms that breach the vaginal epithelial barrier or alter the pH can significantly increase the odds of HIV-1 successfully encountering its target cells.

There is also a strong epidemiological association between concurrent STIs and increased risk of HIV-1 acquisition; in meta-analyses, both human papillomavirus (HPV) and herpes simplex virus 2 (HSV-2) have been associated with a 2- to 3-fold increased risk of HIV-1 acquisition (Freeman et al., 2006; Houlihan et al., 2012). While the precise mechanisms for these epidemiological associations remain unclear, research suggests that the augmented risk is due in part to increased inflammation in the FGT, as shown by higher inflammatory cytokines present in primary genital epithelial cells exposed to co-pathogens (Ferreira et al., 2011), which enhanced HIV-1 infection and replication in target cells. In support of this hypothesis, genital HSV-2 infection has been found to induce a pro-inflammatory profile characterized by increased numbers of HIV-1 target cells that can persist at mucosal sites of HSV-2 reactivation, which may explain in part why individuals with HSV-2 have increased HIV-1 susceptibility (Zhu et al., 2009). In general, any state resulting in the activation of the immune system and/or the recruitment of immune cells, including HIV-1 target cells, may enhance the risk of HIV-1 susceptibility. In fact, a recent study found that the risk of HIV-1 acquisition was increased 3-fold in women with genital inflammation, as defined by elevated HIV-1 target-cell-recruiting chemokines and inflammatory cytokines (Masson et al., 2015). Conversely, reduced inflammation in the FGT was associated with protection from HIV-1. In a cohort of women who were highly exposed to HIV-1 yet remained seronegative (Box 1), dampened genital immunity was proposed to limit target cell availability and activation, and to maintain the protective vaginal barrier, thus decreasing the risk of HIV-1 infecting its target cell population (Lajoie et al., 2012). This relationship has also been demonstrated *in vitro*, where pre-treatment with curcumin, a potent anti-inflammatory agent, prevented the disruption of the mucosal barrier by maintaining ZO-1 and occludin when primary upper FGT cells were exposed to HIV-1 and gp120 (Ferreira et al., 2015). Although an inflammatory milieu is believed to be a primary contributor to HIV-1 susceptibility in women, the frequency of vaginal HIV-1 target cells was found to be the key predictor of HIV-1 susceptibility in a humanized mouse model of heterosexual transmission, independent of inflammatory cytokines (Nguyen et al., 2017). These results suggest that increased numbers of HIV-1 target cells are sufficient to increase susceptibility, even without enhanced pro-inflammatory cytokine production. However, the presence of inflammation significantly increases HIV-1 susceptibility.

As mentioned in the previous section, endogenous sex steroid hormones can modulate immunity in the FGT. Thus, in addition to the integrity of the vaginal barrier and inflammation, sex steroid hormones and, by extension, hormonal contraceptives, represent another factor that can modify susceptibility to HIV-1 in women. Generally, estradiol has been associated with protection against HIV-1, while progesterone and the synthetic progestin DMPA appear to increase risk (Birse et al., 2015a; Morrison et al., 2015; Saba et al., 2013). In fact, some studies have found significantly increased target T cells in women on DMPA (Byrne et al., 2016; Chandra et al., 2013). However, because this observation is not consistent (Michel et al., 2015; Mitchell et al., 2014), it cannot be the sole mechanism by which DMPA enhances susceptibility to HIV-1. Not only are sex hormones and hormonal contraceptives thought to manipulate local HIV-1 target cells, but recent studies have also implicated them in epithelial tissue remodeling, immune cell migration and microbiota composition, suggesting they can impact more than one of the factors that contribute to HIV-1 susceptibility (Achilles et al., 2018; Mitchell et al., 2014; Woods et al., 2018).

**Fig. 3.**
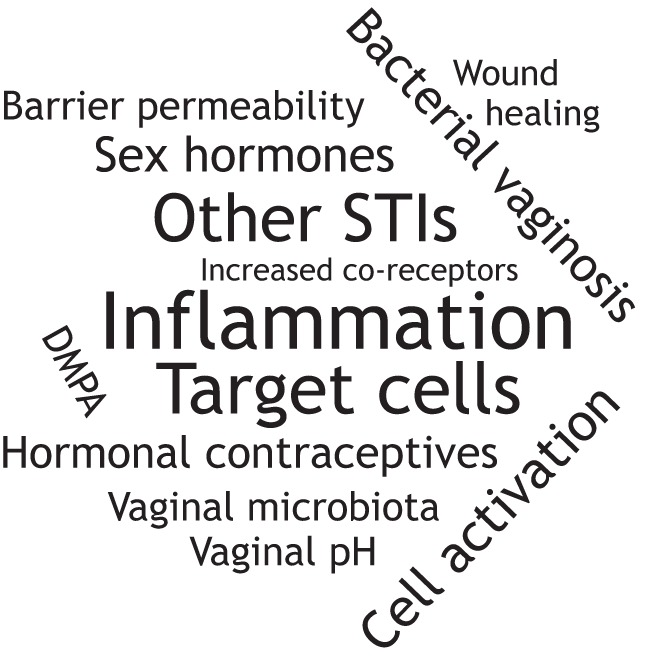
**Factors affecting susceptibility to HIV-1 in women.** There are many factors associated with an increased risk of HIV-1 in women, including disruption of the vaginal barrier, concurrent STIs, the sex hormones, hormonal contraceptives and the vaginal microbiota (VMB). However, the establishment of HIV-1 infection is multifactorial and likely involves mechanisms that remain to be elucidated. A word cloud was created using wordclouds.com to depict factors associated with increasing susceptibility to HIV-1 in women. The size of the text is proportional to the number of publications returned in PubMed (March 2018) when searching for HIV-1 and the corresponding factor. Factors returning 25-99 hits (i.e. hormonal contraceptives) are listed in the smallest font, followed by factors returning 100-499 hits (i.e. sex hormones), 500-999 hits (i.e. target cells), and the largest font represents factors with more than 1000 hits (i.e. inflammation).

A more recently established risk factor for HIV-1 that we highlight in this Review is the VMB. Disruption of the resident VMB can be linked to increased HIV-1 susceptibility, and is most notable in the case of bacterial vaginosis (BV). In fact, BV has been shown to increase the frequency of HIV-1 infection up to 60% (Atashili et al., 2008). Although increased relative abundance of anaerobes and decreased numbers of lactobacilli are key features of BV, the mechanisms by which the VMB increases HIV-1 susceptibility remain largely unknown, but are speculated to relate to their control of vaginal inflammation. Although the etiology and bacterial species that initiate BV are widely debated, it has been proposed that BV might be a biofilm condition (Muzny and Schwebke, 2016; Schwebke et al., 2014). It has been hypothesized that *G. vaginalis*, which can be present in the VMB of women without BV, can induce formation of a biofilm that includes other BV-associated bacteria (Schwebke et al., 2014). The BV biofilm adheres to the vaginal epithelium and produces cytotoxic substances (Muzny and Schwebke, 2016), and may also impact local immune function and thus HIV-1 susceptibility. BV often recurs or is refractive to antibiotic treatment, perhaps due to persistence of the biofilm. It is however important to note that biofilms can be present in women without BV [as assessed by the Amsel criteria (Amsel et al., 1983); Box 1] (Reid, 2018; Swidsinski et al., 2014), and the effect of these biofilms on vaginal immunity is largely unknown. While many factors associated with increasing the risk of HIV-1 in women have been described, including disruption of the vaginal barrier, concurrent STIs, sex hormones/hormonal contraceptives and the VMB, HIV-1 infection is multifactorial and likely involves mechanisms that remain to be elucidated (Fig. 3).

## Effects of the vaginal microbiota on inflammation, immunity and susceptibility to HIV-1

The indigenous microbiota lining mucosal tissues in the human body modulate physiology and immunity at these sites. Although the majority of research demonstrating microbial manipulation of host immune responses has been conducted in the intestinal tract and is reviewed elsewhere (Arranz et al., 2013; Jacobs and Braun, 2014; Round and Mazmanian, 2009), increasing evidence suggests that the VMB plays similar immunomodulatory roles. Unlike the gut microbiome, in which bacterial diversity is associated with health (Kinross et al., 2011), the VMB tends to have low diversity and be predominantly composed of *Lactobacillus* species (Ravel et al., 2011). *Lactobacillus* *crispatus* in particular appears to correlate with protection against STIs and adverse reproductive outcomes via a variety of mechanisms reviewed elsewhere (Anahtar et al., 2018; Mirmonsef and Spear, 2014; Petrova et al., 2015). However, the composition of the VMB varies by ethnicity (Zhou et al., 2007); 80-90% of Caucasian and Asian women and 60% of black and Hispanic women typically have *Lactobacillus*-dominant VMBs (Ravel et al., 2011). This suggests that host genetics might be capable, at least in part, of affecting the bacterial species that colonize the FGT, perhaps via subtle differences in vaginal immunity. It is however important to note that the relationship between the VMB and ethnicity is complex and might rather reflect differences in vaginal hygiene practices, a differential risk of other STIs and/or sexual networks, amongst other factors (Birse et al., 2017; Bradshaw et al., 2014; Brotman et al., 2008; Chaban et al., 2014; Eschenbach et al., 2000; Forcey et al., 2015; Gajer et al., 2012; Marrazzo et al., 2002; Neggers et al., 2007; Schwebke et al., 1999; Vodstrcil et al., 2017; Wessels et al., 2017). Taken together, ethnicity alone is not likely to explain the composition of the VMB in individual women, and a comprehensive review of the factors affecting VMB composition is warranted.

As mentioned previously, certain species of lactobacilli appear to be protective against HIV-1, and prospective studies have shown that women with *Lactobacillus*-dominant VMB are not as likely to acquire HIV-1 as those with more diverse VMB (Gosmann et al., 2017; Low et al., 2011; Nunn et al., 2015). However, the species of *Lactobacillus* seems important, as *Lactobacillus* *iners* did not have as strong of a protective effect against HIV-1 as other species of lactobacilli (Gosmann et al., 2017). The main mechanism by which vaginal bacteria are believed to modify HIV-1 risk is by altering local inflammation and HIV-1 target cell populations (CD4^+^HLA-DR^+^CD38^+^CCR5^+^ cells) (Gosmann et al., 2017; Lennard et al., 2017). At present, it is difficult to discern the precise mechanism by which vaginal bacteria manipulate HIV-1 target cells within the vaginal mucosa. However, *in vitro* bacterial co-culture with VK2/E6E7 vaginal epithelial cells has shown that certain bacterial genera (*Fusobacterium*, *Aerococcus*, *Sneathia*, *Gemella*, *Mobiluncus* and *Prevotella*) induce secretion of pro-inflammatory cytokines, including IL-1α, IL-1β, TNF-α, IL-8 and RANTES, by activating cellular TLRs (Anahtar et al., 2015; Fichorova et al., 2011). Conversely, pro-inflammatory cytokines are not induced when vaginal epithelial cells are co-cultured with *L. crispatus* (considered to be protective against HIV-1) or other vaginal commensals (Box 1) (Doerflinger et al., 2014; Rose et al., 2012), suggesting that epithelial cells sense bacteria via TLRs in a species-specific manner. Clinical studies have demonstrated direct correlations between non-*Lactobacillus-*dominant VMBs and increased levels of inflammatory cyto- and chemokines in the vagina, including TNF-α, IFN-γ, IL-1α, IL-1β, IL-8, IL-10, IL-17, IL-23, MIP-1α and MIP-1β (Anahtar et al., 2015; Gosmann et al., 2017), supporting the *in vitro* results. Genital APCs also sense vaginal microbes. Phenotypic and transcriptional profiling of genital APCs collected from women with highly diverse VMBs demonstrated that APCs likely contribute to genital inflammation by responding via TLR-4, activating NF-κB, inducing chemokine secretion and recruiting HIV-1 target cells (Anahtar et al., 2015). Thus, vaginal bacteria sensed by genital epithelial cells and APCs likely manipulate vaginal inflammation, and can thereby influence susceptibility to HIV-1.

In addition to enhancing HIV-1 target cell recruitment, another mechanism by which vaginal bacteria might enhance susceptibility to HIV-1 is via the breakdown of the vaginal epithelial barrier. As previously mentioned, vaginal bacteria can induce pro-inflammatory cytokines. When primary genital epithelial cells respond to *in vitro* HIV-1 infection, they secrete pro-inflammatory cytokines (TNF-α, IL-6, IL-8, IP-10, RANTES), which results in decreased trans-epithelial resistance (which is a measure of epithelial barrier integrity), disruption of ZO-1 and occludin, and increased leakage of blue dextran dye across the cellular monolayer. This suggests that vaginal bacteria may also impair mucosal barrier function via their induction of pro-inflammatory cytokines (Ferreira et al., 2015; Nazli et al., 2010). Additional evidence supporting the involvement of the VMB in vaginal epithelial barrier impairment can be gleaned from *in vivo* studies linking dysbiosis (Box 1) and increased inflammatory cytokines with altered vaginal proteases and mucosal proteins (Arnold et al., 2016; Borgdorff et al., 2016). This link was stronger during the luteal phase of the menstrual cycle, when progesterone is high (Birse et al., 2015a). Furthermore, impairing the integrity of the vaginal epithelial barrier allows for microbial translocation (Nazli et al., 2010), and this might further perpetuate the recruitment and activation of immune cells, including HIV-1 target cells, above the level that would otherwise occur as a result of bacterial sensing by epithelial and APC TLRs alone. Taken together, the VMB can manipulate susceptibility to HIV-1 by modifying both inflammation and integrity of the FGT barrier.

## Effect of hormones on the vaginal microbiota

Researchers can glean undeniable evidence for the role of sex hormones in shaping the composition of the VMB from studying pubertal girls, women at menopause, animal models and *in vitro* bacterial co-culture. Although the VMB is relatively stable, the major hormonal shifts that occur around puberty (Gerstner et al., 1982; Hickey et al., 2015; Hill et al., 1995) significantly change its composition from mainly anaerobic bacteria (Alvarez-Olmos et al., 2004) to one dominated by lactobacilli. At menopause, when estrogen levels decrease significantly, the VMB is less likely to be dominated by lactobacilli than in pre- and peri-menopausal women. Post-menopausal women were at 7.8-times greater odds of being colonized by a diverse array of bacteria than pre-menopausal women (Brotman et al., 2014b; Shen et al., 2016). Estradiol is thought to be a main driver in shifting the VMB towards *Lactobacillus* dominance, although the mechanisms by which this occurs remain incompletely understood. Estradiol-based hormone replacement therapy (HRT; Box 1) maintains *Lactobacillus* dominance in post-menopausal women (Brotman et al., 2014b; Ginkel et al., 1993; Shen et al., 2016), supporting a link between estradiol and lactobacilli. Additionally, estradiol can increase adhesion of lactobacilli to epithelial cells (Silva et al., 2004), and was proposed to enhance glycogen deposition in the human vaginal epithelium (Cruickshank and Sharman, 1934; Farage and Maibach, 2006), via an unknown mechanism. Glycogen, a glucose polysaccharide and an important nutrient for lactobacilli (Cruickshank and Sharman, 1934; Mirmonsef et al., 2014; Paavonen, 1983), can be synthesized by vaginal epithelial cells and released into the vaginal mucus (Fig. 1). Experimental administration of estradiol can induce glycogen deposition in vaginal tissues in hamsters and non-human primates (Gregoire and Parakkal, 1972; Gregoire and Richardson, 1970) via an unknown mechanism. The availability of glycogen in the epithelial cells is thought to select for vaginal colonization by microbes capable of metabolizing it. However, the relationship between estradiol, free glycogen released into the vaginal lumen by the epithelial cells and lactobacilli may not be as simple, as recent studies did not find a direct correlation between circulating peripheral estradiol and free glycogen (Mirmonsef et al., 2016; Mirmonsef and Spear, 2014), and few, if any, species of vaginal lactobacilli directly metabolize glycogen (Martín et al., 2008; Nunn and Forney, 2016; Spear et al., 2014; Stewart-Tull, 1964). The lack of correlation between estradiol and free glycogen could be due to the fact that estradiol concentrations fluctuate rapidly over the menstrual cycle, peripheral estradiol concentrations are generally lower than those in the FGT, and free glycogen might be differentially utilized or degraded depending on the composition of the VMB (Mirmonsef et al., 2016). Furthermore, α-amylase, an enzyme that cleaves glycogen into simple sugars, has been isolated in fluid from the lower FGT (Spear et al., 2014). This suggests that, rather than metabolizing glycogen, certain bacterial species in the VMB might directly use the simple sugars as substrates. Nevertheless, high free glycogen is associated with a *Lactobacillus*-dominant VMB (Mirmonsef et al., 2014), and a clear relationship exists between estradiol and vaginal lactobacilli in post-menopausal women on HRT (Brotman et al., 2014b; Shen et al., 2016), albeit via incompletely understood mechanisms.

Although endogenous hormones can shape the VMB, we are only beginning to explore the modulation of the vaginal bacteria by hormonal contraceptives (Achilles and Hillier, 2013; Brotman et al., 2014a). This is a timely and controversial topic, due to the potential for hormone-VMB interactions to manipulate HIV-1 target cells in the reproductive tract mucosa. Meta-analyses show that women on DMPA are 40% more likely to acquire HIV-1 than women that do not use hormonal contraceptives (Morrison et al., 2015; Polis et al., 2016). In 2015, 56 million women worldwide used injectable hormonal contraceptives (United Nations Department of Economic and Social Affairs Population Division, 2015). Therefore, understanding their impact on vaginal microbes is of great importance. Indeed, a recent study found that the strongest independent predictors of genital inflammation were use of hormonal contraceptives (all methods grouped) and a VMB subtype that predominantly included women with high Nugent scores (indicating high microbial diversity) (Lennard et al., 2017). These findings suggest that both hormonal contraceptives and the vaginal microbes are able to modify HIV-1 target cells in the vaginal mucosa. Previous studies that only examined a targeted subset of the VMB and did not employ 16S rRNA gene sequencing (Box 1) did not find use of hormonal contraceptives to be a confounder of VMB composition (Borgdorff et al., 2015), and showed that women on hormonal contraceptives were more likely to have *Lactobacillus* *fermentum* in their VMB than those who were not (Kazi et al., 2012). Moreover, the initiation of DMPA injections decreased *G. vaginalis* and total bacterial load (Roxby et al., 2016), and it also reduced the proportion of women harboring H_2_O_2_-producing *Lactobacillus* (Mitchell et al., 2014). Additionally, a quantitative-PCR-based study quantified five *Lactobacillus* and four BV-associated species to demonstrate that DMPA was associated with lower quantities of lactobacilli compared to women not using hormonal contraceptives, even excluding BV as a potential confounder (Jespers et al., 2017). Although the aforementioned studies included women on a variety of systemically administered hormonal contraceptives such as injectables, oral pills, patches, etc., emerging data suggests that estrogen-containing vaginal rings, which are topically administered, may have a more profound effect on vaginal lactobacilli due to their proximity in the vaginal tract (De Seta et al., 2012; Hardy et al., 2017; Huang et al., 2015; Veres et al., 2004). Thus, the route of administration of hormonal contraceptives is likely to differentially impact the VMB, and this should be kept in mind for multi-purpose HIV-1 prevention strategies aimed at combining antiretroviral therapy and hormonal contraceptives.

**Fig. 4.**
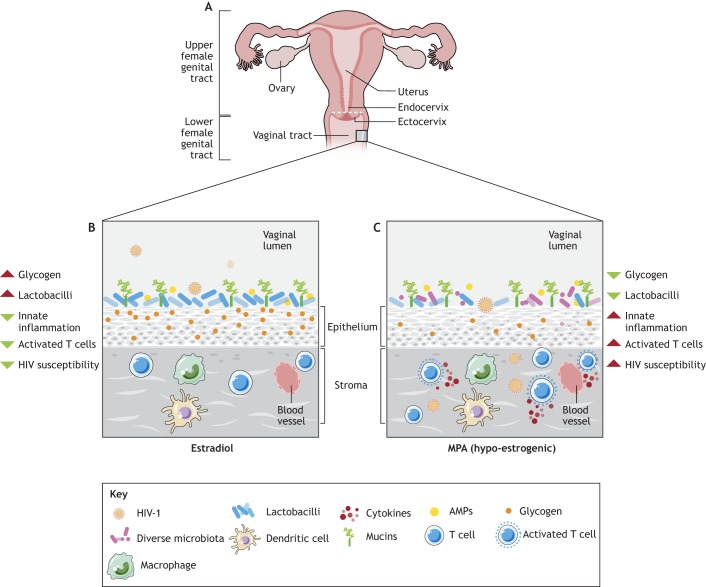
**The sex-hormone–microbiome–immunity axis and HIV-1 susceptibility in women.** An illustration depicting how the hormonal milieu of the lower female genital tract (FGT) might impact susceptibility to HIV-1. (A) Anatomy of the FGT. (B,C) Immunity and the microbiota in the lower FGT. (B) When estradiol is dominant, glycogen, a glucose polysaccharide that is correlated with enhanced vaginal lactobacilli, is abundant and adequately sustains the colonization of the vaginal mucosa by *Lactobacillus* species, via unknown mechanisms. Several studies have reported that a vaginal microbiota (VMB) dominated by species of lactobacilli is protective against HIV-1, perhaps a result of dampened innate inflammation and a reduction in activated T cells. In a microenvironment influenced by estradiol, epidemiological and experimental studies collectively suggest that susceptibility to HIV-1 appears to be hampered (Hapgood et al., 2018). (C) Conversely, when DMPA (MPA is the active ingredient in DMPA) is dominant, endogenous estradiol is suppressed due to the hypo-estrogenic effect of DMPA. As a result, glycogen deposition in the vaginal epithelium may be minimized, depleting one of the important nutrients that may sustain the protective vaginal lactobacilli. Given the depletion in nutrients, lactobacilli are not as numerous as when they are under the influence of estradiol, and a more diverse array of bacteria is subsequently supported in the VMB. In turn, the diverse bacteria might induce innate immunity in the FGT, upregulating cytokines, inflammation and activated T cells. Given that activated T cells are a major target of HIV-1, this type of environment would ultimately enhance susceptibility to HIV-1 in women. At present, it is unclear whether this proposed mechanism is a direct result of exposure to DMPA or an indirect result of hypo-estrogenism. AMPs, antimicrobial peptides; DMPA, depot-medroxyprogesterone acetate; HIV-1, human immunodeficiency virus type 1; MPA, medroxyprogesterone acetate.

Next-generation 16S rRNA gene sequencing of the VMB demonstrated that VMBs from individual women do not cluster together by method of contraception (Birse et al., 2017; Byrne et al., 2016), suggesting that, although hormonal contraceptives did not cause major changes in the VMB, subtle changes may be biologically relevant. Indeed, a recent 16S rRNA sequencing study found that women on oral contraceptives had enhanced *L. crispatus* and decreased BV-associated bacteria loads (Brooks et al., 2017), supporting the findings of meta-analyses showing that hormonal contraceptives can reduce the risk of BV by up to 30% (van de Wijgert et al., 2013; Vodstrcil et al., 2013). As many of the prior studies did not consider the specific method of (hormonal) contraception (i.e. grouping all contraception or all progestins) or the duration of hormonal contraceptive treatment as independent variables, and did not account for the potential confounding effect of including women with BV, the true effect of hormonal contraceptives on the VMB has been difficult to discern. To address this, our group is conducting 16S rRNA gene sequencing in a comprehensive cross-sectional clinical study where we control for many potential confounders. We aimed to examine the effect of hormonal contraceptives on VMB alpha-diversity (Box 1) and the vaginal microenvironment. We thought that this was particularly warranted given recent publications, which demonstrated that women with non-*Lactobacillus*-dominant VMB were at greater risk of HIV-1 infection (Gosmann et al., 2017), and which identified hormonal contraceptives and a diverse VMB as the strongest predictors of genital inflammation (Lennard et al., 2017). Taken together, we propose that one of the mechanisms by which DMPA enhances susceptibility to HIV-1 may result from its hypo-estrogenic effect. In the absence of estradiol, the vaginal epithelium and/or mucus may become depleted of *Lactobacillus*-promoting glycogen, which subsequently allows a diverse array of bacteria to colonize. Consequently, this diverse microbiota might enhance mucosal inflammation and/or HIV-1 target cell activation, and thus enhance susceptibility to HIV-1 infection. A summary of how DMPA might modulate the VMB and susceptibility to HIV-1 in women is presented in Fig. 4.

## Conclusions

Herein, we have summarized the current literature and our view of how the sex-hormone–microbiome–immunity axis has the potential to affect HIV-1 susceptibility in women. Factors that enhance inflammation/inflammatory cytokines, and thus HIV-1 target cells, in the FGT can modify the risk of infection. Factors that affect the integrity of the protective epithelial barrier in the FGT can also modify the risk of HIV-1 infection by allowing viral particles to access target cells more readily. Although a relatively recent finding, factors that modify the vaginal microbiota are also believed to be able to modify HIV-1 risk via their impact on both inflammation and barrier integrity. Moving forward, rigorous clinical and preclinical studies aimed at examining how hormonal contraceptives affect the vaginal microbiota are critically needed. Understanding the factors that influence VMB composition, and the mechanisms by which these bacteria modify host factors, including the population of HIV-1 target cells, may help in understanding why women using DMPA as a contraceptive are up to 40% more likely to acquire HIV-1 than women not using hormonal contraceptives (Gosmann et al., 2017).
